# Resampling Multi-Resolution Signals Using the Bag of Functions Framework: Addressing Variable Sampling Rates in Time Series Data

**DOI:** 10.3390/s25154759

**Published:** 2025-08-01

**Authors:** David Orlando Salazar Torres, Diyar Altinses, Andreas Schwung

**Affiliations:** Department of Automation Technology and Learning Systems, South Westphalia University of Applied Sciences, 59494 Soest, Germany; altinses.diyar@fh-swf.de (D.A.); schwung.andreas@fh-swf.de (A.S.)

**Keywords:** Bag of Functions framework, multi-resolution signals, resampling, time-invariant methods, time series decomposition, variable sampling rates

## Abstract

In time series analysis, the ability to effectively handle data with varying sampling rates is crucial for accurate modeling and analysis. This paper presents the MR-BoF (Multi-Resolution Bag of Functions) framework, which leverages sampling-rate-independent techniques to decompose time series data while accommodating signals with differing resolutions. Unlike traditional methods that require uniform sampling frequencies, the BoF framework employs a flexible encoding approach, allowing for the integration of multi-resolution time series. Through a series of experiments, we demonstrate that the BoF framework ensures the precise reconstruction of the original data while enhancing resampling capabilities by utilizing decomposed components. The results show that this method offers significant advantages in scenarios involving irregular sampling rates and heterogeneous acquisition systems, making it a valuable tool for applications in fields such as finance, healthcare, industrial monitoring, IoT networks, and sensor networks.

## 1. Introduction

The processing and analysis of signals acquired at varying sampling rates present fundamental challenges across multiple scientific and engineering disciplines, including biomedical monitoring, environmental sensing, industrial diagnostics, IoT networks, and sensor networks. The variability in sampling rates arises due to differences in acquisition hardware, adaptive sensing strategies, and application-specific requirements. For instance, mobile health monitoring devices often adjust acquisition frequencies to optimize battery life, while environmental sensors might increase acquisition frequency during critical events [1]. Standardizing these multi-resolution signals via resampling techniques facilitates cross-comparison, integration, and coherent analysis of heterogeneous datasets [2]. However, resampling introduces challenges, including spectral distortion, aliasing, and phase misalignment, which can significantly affect signal integrity and downstream processing tasks [3,4].

A promising approach for signal modeling and reconstruction is the Bag of Functions (BoF) framework. BoF represents time series as a linear combination of continuous basis functions, such as sinusoids, linear polynomials, step functions, Gaussians, or exponentials, transforming discrete samples into a continuous time signal representation [5,6]. This enables seamless resampling at any resolution, mitigates interpolation artifacts like aliasing, and provides interpretable signal components through a compact parametric form. Unlike classical methods, BoF is data-driven and adaptive, capturing the signal’s underlying structure without rigid sampling assumptions. However, existing BoF approaches assume uniform sampling across the dataset, which limits their applicability in common real-world scenarios involving heterogeneous sampling rates.

In this paper, we propose an adaptive decomposition framework that represents and reconstructs signals without direct interpolation. Our approach decomposes signals into interpretable continuous components while preserving both spectral and temporal characteristics across arbitrary sampling schemes. Instead of interpolating to a fixed grid, we build a functional representation that allows for direct resampling and reconstruction at any desired resolution. This enables seamless integration of signals with different sampling rates and facilitates joint analysis across heterogeneous sources. Our key contributions can be summarized as follows:

1. We develop an extension of the Bag of Functions approach to process data collected at varying sampling rates.
2. We propose a scalable decomposition framework based on the Bag of Functions that enables the representation of discrete signals into continuous functions, ensuring the preservation of spectral and temporal characteristics.
3. We introduce an unsupervised resampling mechanism that standardizes time series with different sampling rates, allowing for a unified representation that facilitates joint analysis and data fusion.
4. We validate our approach on one synthetic and three real-world datasets, demonstrating its effectiveness in reconstructing signals while preserving statistical and spectral properties across different sampling rates.

The rest of this paper is organized as follows. Section 2 provides a comprehensive review of existing literature, highlighting the theoretical and practical gaps that motivate our contribution. Section 3 details the theoretical basis of the BoF framework, including its machine learning architecture and our proposed technique for handling multi-resolution time series data. Section 4 presents the experimental setup and discusses its performance on synthetic and real-world datasets. Finally, Section 5 summarizes the main findings and proposes possible future research.

## 2. Related Work

This section examines two research areas fundamental to our work: time series decomposition techniques and multi-resolution signal processing methods. Time series decomposition approaches extract constituent components, revealing essential patterns for analysis. Multi-resolution signal processing addresses the challenges of heterogeneous sampling rates, enabling the unification of signals acquired under varying temporal conditions. These research fields form the base of our proposed Bag of Functions framework.

### 2.1. Time Series Decomposition

Time series decomposition separates a signal into components such as trend, seasonality, and noise to facilitate analysis. Classical methods, including Seasonal–Trend decomposition using LOESS [7] and ARIMA-based approaches [8,9], are widely used for regularly sampled data due to their effectiveness in structured time series. However, they assume a fixed sampling rate and rely on rigid assumptions about periodicity and stationary, limiting their applicability to irregularly sampled data.

Frequency-domain methods, such as the Fourier and wavelet transforms, introduced multi-scale analysis and are fundamental tools for signal decomposition [10]. However, their application to non-uniformly sampled data requires specialized adaptations. Methods such as non-uniform discrete Fourier transform [11] and adaptive wavelet approaches [12] attempt to address these limitations, yet challenges remain in preserving spectral properties under varying sampling densities.

Empirical Mode Decomposition (EMD) [13] and its variants (e.g., Ensemble EMD, CEEMDAN) iteratively decompose complex, non-linear, and non-stationary signals into a set of intrinsic mode functions, enabling localized time–frequency analysis without requiring a predefined basis [14]. Numerous studies have demonstrated their effectiveness in practical applications, including biomedical signal analysis, fault diagnosis, and environmental data processing [15]. However, EMD can be sensitive to sampling irregularities, often requiring ensemble-based extensions to improve robustness [14].

Recent machine learning innovations have transformed decomposition. Supervised methods, particularly Long Short-Term Memory networks, excel at capturing long-term dependencies, making them ideal for modeling trends and seasonal patterns [16,17]. Nevertheless, these models operate under the assumption of uniform sampling rates. Hybrid models have also emerged, combining machine learning techniques with traditional statistical approaches to improve precision [18,19,20], while residual decomposition architectures further enhance robustness by iteratively refining each component to produce a stable representation of the original signal [6].

Machine learning methods offer an alternative to traditional decomposition techniques, enabling automated feature extraction and capturing non-linear patterns. However, processing time series data with multiple sampling rates remains a critical challenge. To tackle this, we extend the Bag of Functions framework for time series decomposition, incorporating an adaptive approach that allows for the representation and analysis of signals with heterogeneous sampling frequencies.

### 2.2. Multi-Resolution Signal Processing Methods

Multi-resolution signal processing provides a theoretical foundation for analyzing time series data collected at different sampling rates, a critical aspect of integrating heterogeneous signals for decomposition [21]. Classical resampling and interpolation techniques remain central to this field due to their straightforward conceptual basis and ability to standardize signals effectively in many practical scenarios [22].

Traditional methods rely on simple interpolation strategies, such as connecting data points with straight lines or fitting smooth polynomial curves to approximate signal behavior between samples. These techniques are valued for their ability to align regularly sampled data with minimal complexity, making them enduring tools for basic multi-resolution tasks [12]. Frequency-domain resampling, which adjusts signal representations using spectral transformations, offers another classical approach by preserving key frequency components during rate conversion [23]. More advanced filtering methods, based on finite impulse response principles, further refine this process by reducing aliasing and enhancing signal fidelity [24].

These classical techniques persist because they provide a reliable framework for handling uniform or mildly irregular signals, balancing theoretical simplicity with practical utility in fields like audio analysis and temporal data alignment. However, these approaches face significant limitations when applied to time series with highly variable sampling rates [25]. Linear or polynomial interpolation often assumes signal continuity that may not hold, introducing distortions, while frequency-based methods struggle to adapt to non-uniform sampling densities without compromising spectral integrity [26]. Recent theoretical developments have explored adaptive multi-resolution frameworks to address these challenges, focusing on capturing temporal patterns across diverse scales without relying on rigid pre-processing [27]. Such approaches enhance flexibility by modeling signals at multiple resolutions simultaneously, yet they often prioritize specific use cases, like prediction, over general resampling and decomposition needs, leaving gaps in handling arbitrary sampling frequencies.

To address these shortcomings, we propose to transform signals into a functional space where they are represented as continuous functions, rather than discrete points or frequency bands. This enables seamless resampling to any target frequency while preserving the signal’s intrinsic properties, overcoming the artifacts and assumptions inherent in classical and modern discrete methods.

## 3. Bag of Functions for Resampling Multi-Resolution Signals

Following the analysis of related work in Section 2, we now present our proposed method for multi-resolution signal resampling, designed to address the limitations identified in prior approaches. Therefore, we begin by defining the problem of resampling multi-resolution signals within the Bag of Functions framework. We then introduce our methodology for reconstructing signals sampled at different frequencies and aligning them to a common target rate. The section begins with a description of the neural network architecture, followed by our proposed approach for ensuring a coherent signal representation across different sampling rates.

### 3.1. Problem Definition

Let $D={\left\{\left(x_{i},f_{i}\right)\right\}}_{i=1}^{N}$ denote a dataset of *N* multivariate time series signals, where each signal $x_{i}\in R^{L_{i}\times q}$ is sampled at frequency $f_{i}$, resulting in $L_{i}$ discrete samples of *q* sensors. Each discrete signal $x_{i}$ represents samples from an underlying continuous function $x_{i}\left(t\right)$ such that $x_{i}\left[n\right]=x_{i}\left(nT_{i}\right)$, where $T_{i}=1/f_{i}$ is the sampling period. The objective is to obtain from the discrete samples $x_{i}\left[n\right]$ a continuous representation ${\hat{x}}_{i}\left(t\right)$ that allows resampling at a common target frequency $f^{*}$. This requires finding a transformation H that can adapt to different sampling frequencies $f_{i}$ and generate a consistent continuous representation ${\hat{x}}_{i}\left(t\right)=H\left(x_{i}\right)$. The transformation H must process signals with diverse temporal resolutions, correctly aligning information in the time domain while preserving their fundamental characteristics across different time scales. This continuous signal ${\hat{x}}_{i}\left(t\right)$ enables obtaining the desired discrete signal $x_{i}^{*}\left[m\right]={\hat{x}}_{i}\left(mT^{*}\right)$ with the target sampling period $T^{*}=\frac{1}{f^{*}}$ represented as a vector $x_{i}^{*}$ with the dimensions $x_{i}^{*}\in R^{L_{i}^{*}\times q}$. To ensure that the transformation and resampling minimize distortion, the optimization objective is to minimize the loss L that evaluates the reconstruction quality and information preservation:

(1) $$\min_{H}\sum_{i=1}^{N}L\left(x_{i},H\left(x_{i}\right)\left[n\right]\right).$$

### 3.2. Neural Network Architecture

In this study, we investigate a model architecture designed for the structured decomposition of time series data into its key components while intentionally omitting the stochastic noise. Noise introduces irregular, non-structured fluctuations in the signal, which can mislead the model during resampling across different sampling rates.

**Figure 1:**
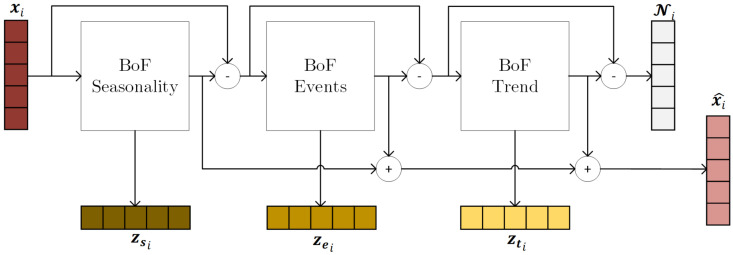
The residual Bag of Functions architecture [28].

The architecture follows a residual design, which incrementally extracts and reconstructs these interpretable signal components using specialized neural submodules. This hierarchical decomposition is achieved through the use of multiple Bag of Functions blocks, as illustrated in Figure 1.

The signal reconstruction proceeds in three BoF submodels. Each BoF block employs the residual information from previous submodels:

(2) $$\begin{array}{cc} {\hat{x}}_{s_{i}} & ={\mathrm{BoF}}_{s}\left(x_{i}\right) \end{array}$$

(3) $$\begin{array}{cc} {\hat{x}}_{e_{i}} & ={\mathrm{BoF}}_{e}\left(x_{i}-{\hat{x}}_{s_{i}}\right) \end{array}$$

(4) $$\begin{array}{cc} {\hat{x}}_{t_{i}} & ={\mathrm{BoF}}_{t}\left(x_{i}-{\hat{x}}_{s_{i}}-{\hat{x}}_{e_{i}}\right) \end{array}$$

**Figure 2:**
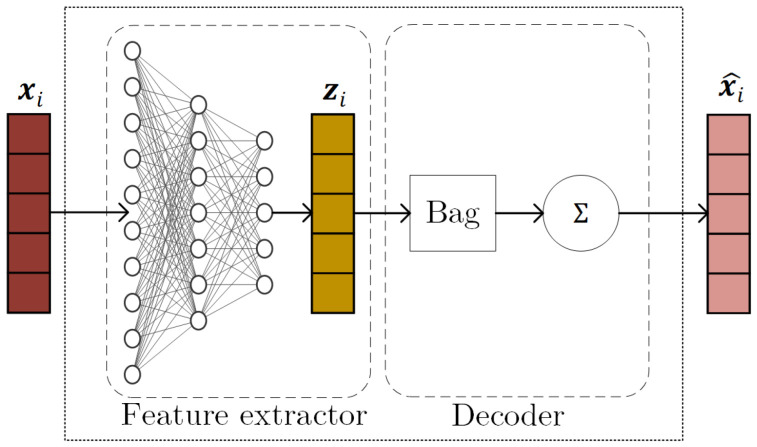
The Bag of Functions core block [28].

This Bag of Functions approach [5], forms the foundation of the model. It enables the neural network to learn interpretable representations by parameterizing basis functions that are then used to synthesize the signal. The base BoF architecture is presented in Figure 2.

A fundamental BoF block operates by first extracting features from an input vector $x_{i}$ using a feature extractor $f_{\theta }$. This process yields a latent parameter vector, $z_{i}=f_{\theta }\left(x_{i}\right)$, which then parameterizes a predefined collection of *A* basis functions, denoted as ${\left\{\phi_{a}\right\}}_{a=1}^{A}$. Finally, the estimated component, ${\hat{x}}_{i}$, is reconstructed by summing these parameterized functions over a given time interval vector t, as follows:

(5) $$\begin{array}{c} {\hat{x}}_{i}=\sum_{a=1}^{A}\phi_{a}\left(t;z_{i,a}\right). \end{array}$$

Here, the feature extractors $E_{\theta_{s}}$, $E_{\theta_{e}}$, and $E_{\theta_{t}}$ generate latent representations $z_{s_{i}}$, $z_{e_{i}}$, and $z_{t_{i}}$, respectively, each of which parameterizes a distinct set of basis functions. These basis functions $\phi_{j}$, $\phi_{k}$, and $\phi_{l}$ span the space of seasonality, events, and trends.

$$\begin{array}{cc} z_{s_{i}}=E_{\theta_{s}}\left(x_{i}\right)\; & \mathrm{and}\;{\hat{x}}_{s_{i}}=\sum_{j=1}^{J}\phi_{j}\left(t;z_{s_{i,j}}\right)\\ z_{e_{i}}=E_{\theta_{e}}\left(x_{i}-{\hat{x}}_{s_{i}}\right)\; & \mathrm{and}\;{\hat{x}}_{e_{i}}=\sum_{k=1}^{K}\phi_{k}\left(t;z_{e_{i,k}}\right)\\ z_{t_{i}}=E_{\theta_{t}}\left(x_{i}-{\hat{x}}_{s_{i}}-{\hat{x}}_{e_{i}}\right)\; & \mathrm{and}\;{\hat{x}}_{t_{i}}=\sum_{l=1}^{L}\phi_{l}\left(t;z_{t_{i,l}}\right) \end{array}$$

The final reconstructed signal is obtained by summing the outputs of the three BoF blocks:

(6) $${\hat{x}}_{i}=\sum_{j=1}^{J}\phi_{j}\left(t;z_{s_{i,j}}\right)+\sum_{k=1}^{K}\phi_{k}\left(t;z_{e_{i,k}}\right)+\sum_{l=1}^{L}\phi_{l}\left(t;z_{t_{i,l}}\right).$$

The Bag of Functions approach models signals by parameterizing a predefined set of basis functions, allowing the network to synthesize complex temporal patterns from interpretable components. The number of basis functions directly governs the model’s representational capacity. Increasing this number enhances flexibility and expressive power, but also leads to greater computational cost during both training and inference. Consequently, the selection of basis functions becomes a critical hyperparameter in the BoF framework, analogous to architectural choices in traditional neural networks. Importantly, this selection need not be arbitrary; incorporating domain knowledge can guide the design or restriction of the basis function set, yielding more efficient models that retain high performance while reducing redundancy and computational overhead.

This formulation allows the model to synthesize time series data via a composition of smooth, interpretable basis functions. The explicit exclusion of the noise component ensures that the reconstruction focuses solely on the structured patterns within the signal. Algorithm 1 presents the optimization procedure for the Residual Bag of Functions model with function parameterization.

**Algorithm 1** Optimization of Residual BoF Model with function parametrization.**Require:** Dataset $D={\left\{x_{i},f_{i}\right\}}_{i=1}^{N}$, basis function families ${\left\{\phi_{j}\right\}}_{j=1}^{J}$, ${\left\{\phi_{k}\right\}}_{k=1}^{K}$, ${\left\{\phi_{l}\right\}}_{l=1}^{L}$, learning rate η, number of epochs *M*
  1:Initialize parameters $\theta_{s},\theta_{e},\theta_{t}$  2:
**Repeat until convergence:**
  3:**for** t=1 to *M* **do**  4:  **for** each sample $(x_{i},t_{i})\in D$ **do**                      ▹ **1. Seasonality Component**  5:          $z_{s_{i}}\leftarrow E_{\theta_{s}}\left(x_{i}\right)$  6:          ${\hat{x}}_{s_{i}}\leftarrow \sum_{j=1}^{J}\phi_{j}\left(t_{i};z_{s_{i,j}}\right)$
▹**2. Event Component (residual)**
  7:          $r_{e_{i}}\leftarrow x_{i}-{\hat{x}}_{s_{i}}$  8:          $z_{e_{i}}\leftarrow E_{\theta_{e}}\left(r_{e_{i}}\right)$  9:          ${\hat{x}}_{e_{i}}\leftarrow \sum_{k=1}^{K}\phi_{k}\left(t_{i};z_{e_{i,k}}\right)$
▹**3. Trend Component (second residual)**
10:          $r_{t_{i}}\leftarrow x_{i}-{\hat{x}}_{s_{i}}-{\hat{x}}_{e_{i}}$11:          $z_{t_{i}}\leftarrow E_{\theta_{t}}\left(r_{t_{i}}\right)$12:          ${\hat{x}}_{t_{i}}\leftarrow \sum_{l=1}^{L}\phi_{l}\left(t_{i};z_{t_{i,l}}\right)$
▹**4. Signal Reconstruction and Loss Computation**
13:          ${\hat{x}}_{i}\leftarrow {\hat{x}}_{s_{i}}+{\hat{x}}_{e_{i}}+{\hat{x}}_{t_{i}}$
14:          $L_{i}\leftarrow {∥{\hat{x}}_{i}-x_{i}∥}_{2}^{2}$15:  **end for**                                  ▹ **5. Parameter Update**16:  $\theta_{s}\leftarrow \theta_{s}-\eta \cdot \nabla_{\theta_{s}}\sum_{i=1}^{N}L_{i}$17:  $\theta_{e}\leftarrow \theta_{e}-\eta \cdot \nabla_{\theta_{e}}\sum_{i=1}^{N}L_{i}$18:  $\theta_{t}\leftarrow \theta_{t}-\eta \cdot \nabla_{\theta_{t}}\sum_{i=1}^{N}L_{i}$19: **end for**20: **return** Trained parameters $\theta_{s},\theta_{e},\theta_{t}$


In the subsequent section, we address the adaptation of this model to input signals with varying sampling rates.

### 3.3. Resampling Strategy Within the Bag of Functions Framework

To effectively process time series with varying sampling frequencies within the Bag of Functions framework, we implement a strategy that combines interleaved padding and masking. This approach ensures that all input sequences are consistently represented at a target length, thereby facilitating uniform processing by the neural network.

To accommodate signals of different lengths and frequencies, we transform each input time series $x_{i}\in R^{L_{i}}$ into a tensor $x_{i}^{\mathrm{pad}}\in R^{L_{\mathrm{target}}}$ of a predefined target length $L_{\mathrm{target}}=D\cdot f^{*}\in N$, where *D* is the maximum duration of the signals in the dataset and $f^{*}$ is the desired target sampling frequency. This is achieved through an interleaved padding process, where the original samples are placed in the tensor at positions corresponding to their original sampling times, and the remaining positions are filled with zeros.

Specifically, for each sample $x_{i}$, we calculate the position $p_{n}$ of each sample $x_{i}\left[n\right]$ in the padded tensor using the following formula:

(7) $$p_{n}=\left\lfloor \frac{n}{f_{i}}\cdot \frac{L_{\mathrm{target}}}{D}+\frac{1}{2}\right\rfloor$$

where $n\in \{0,\ldots ,L_{i}-1\}$ is the sample index, $f_{i}$ is the sampling frequency, and *D* is the maximum duration of the signal. The padded sequence $x_{i}^{\mathrm{pad}}\in R^{L_{\mathrm{target}}}$ is then constructed such that

(8) $$x_{i}^{\mathrm{pad}}\left[m\right]=\left\{\begin{array}{cc} x_{i}\left[n\right] & \mathrm{if}\;m=p_{n}\;\mathrm{for}\;\mathrm{some}\;n\in \left\{0,\ldots ,L_{i}-1\right\}\\ 0 & \mathrm{otherwise} \end{array}\right.$$

To prevent the network from learning the padded elements, we generate a binary mask $\sigma_{i}\in {\left\{0,1\right\}}^{L_{\mathrm{target}}}$ that indicates the positions of the original samples. This mask is defined as follows:

(9) $$\sigma_{i}\left[m\right]=\left\{\begin{array}{cc} 1 & \mathrm{if}\;m\in \{p_{0},\ldots ,p_{L_{i}-1}\}\\ 0 & \mathrm{otherwise} \end{array}\right.$$

During the computation of the loss function L, we apply this mask $\sigma_{i}$ to the reconstructed output ${\hat{x}}_{i}\in R^{L_{\mathrm{target}}}$ to ensure that the optimization process focuses only on the original data points:

(10) $$L\left(x_{i},{\hat{x}}_{i}\right)={∥x_{i}^{\mathrm{pad}}-\sigma_{i}⊙{\hat{x}}_{i}∥}_{2}^{2}$$

where ⊙ denotes element-wise multiplication and ${∥\cdot ∥}_{2}$ is the Euclidean norm. This masking strategy ensures that the network learns to reconstruct the original signal without being influenced by the padded zeros.

In cases where $L_{i}>L_{\mathrm{target}}$, we apply downsampling by a factor $d=\lfloor L_{i}/L_{\mathrm{target}}\rfloor$. The downsampled signal $x_{i}^{\mathrm{down}}\in R^{L_{\mathrm{target}}}$ is defined as follows:

(11) $$x_{i}^{\mathrm{down}}\left[m\right]=x_{i}\left[md\right],\;\forall m\in \left\{0,\ldots ,L_{\mathrm{target}}-1\right\}.$$

The mask $\sigma_{i}$ for downsampled signals is a vector of ones:

(12) $$\sigma_{i}\left[m\right]=1,\;\forall m\in \left\{0,\ldots ,L_{\mathrm{target}}-1\right\}.$$

This combined approach of interleaved padding, downsampling, and masking allows the network to effectively extract continuous time series signals with diverse sampling rates, ensuring consistent input dimensions and focused learning on the original characteristics.

## 4. Evaluation

Having detailed our multi-resolution resampling approach in Section 3, we now evaluate its performance against conventional methods using the diverse datasets to assess the effectiveness of the proposed reconstruction and resampling method. Therefore, we first provide a detailed description of the dataset used in our experiments, emphasizing its key characteristics. Next, we outline the experimental setup, specifying the training and evaluation protocols. Finally, we present the results and conduct a thorough analysis of the method’s performance.

### 4.1. Synthetic Data Generation

To generate synthetic time series, we follow the methodology proposed in [28], where each signal $x_{i}$ is constructed as the sum of four fundamental components: trend, seasonality, event, and noise. The additive model is defined as follows:

(13) $$x_{i}\left(t\right)=s_{i}\left(t\right)+e_{i}\left(t\right)+t_{i}\left(t\right)+n_{i}\left(t\right).$$

The parameters of each component are randomly sampled from uniform distributions:

1. Seasonality: Two sets of sinusoidal functions are used:
  (14) $$s_{i}\left(t\right)=\sum_{j=1}^{2}a_{1}^{\left(j\right)}\sin\left(2\pi a_{2}^{\left(j\right)}t+a_{3}^{\left(j\right)}\right),$$
  with parameters sampled as $a_{1}^{\left(1\right)}∼U\left(2,3\right)$, $a_{2}^{\left(1\right)}∼U\left(3,5\right)$, $a_{3}^{\left(1\right)}∼U\left(0,2\pi \right)$ and $a_{1}^{\left(2\right)}∼U\left(3,4\right)$, $a_{2}^{\left(2\right)}∼U\left(1,2\right)$, $a_{3}^{\left(2\right)}∼U\left(0,2\pi \right)$.
2. Trend: The trend component is modeled as a linear function
  (15) $$t_{i}\left(t\right)=b_{1}t+b_{2},$$
  where the parameters are sampled as $b_{1}∼U\left(-2,2\right)$ and $b_{2}∼U\left(-2,2\right)$.
3. Event: This is defined as a Gaussian function
  (16) $$e_{i}\left(t\right)=e_{1}\exp\left(-\frac{{\left(t-e_{2}\right)}^{2}}{2e_{3}^{2}}\right),$$
  where the parameters are sampled as $e_{1}∼U\left(-1,1\right)$, $e_{2}∼U\left(0,1\right)$, $e_{3}∼U\left(0.5,1\right)$.
4. Noise: The noise component is modeled as a uniform distribution
  (17) $$n_{i}\left(t\right)∼U\left(-0.5,0.5\right).$$

**Figure 3:**
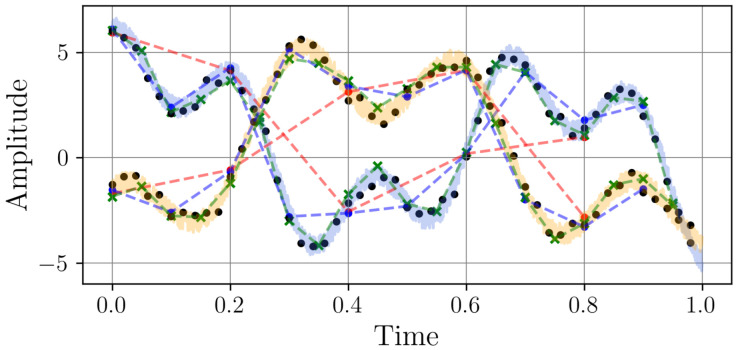
Two samples from the time series dataset with different sampling rates. The continuous light-colored (blue and yellow) signal shows the original time series sampled at 2 kHz. The markers, connected by dashed lines, represent the signal sampled to different frequencies: red (5 Hz), blue (10 Hz), green (20 Hz), and black (50 Hz).

The dataset is constructed by sampling the previously defined continuous signals $x_{i}\left(t\right)$ at different frequencies. Each discrete signal $x_{i}={\left\{x_{i}\left[n\right]\right\}}_{n=0}^{L_{i}-1}$ consists of $L_{i}$ samples, where the sampling period is $T_{i}=1/f_{i}$. A total of 1000 time series were generated per sampling frequency, with 80% used for training and 20% for validation. The selected sampling frequencies are 5, 10, 20, 50, 100, and 500 Hz. Since all signals have a fixed duration of one second, the number of samples per signal varies accordingly. The resulting dataset consists of time series with different resolutions, as illustrated in Figure 3.

The samples exhibit a smooth, periodic oscillation with consistent peaks and troughs. This regularity suggests a well-defined, predictable pattern, making it ideal for testing resampling methods on structured signals. Together, these samples create a balanced dataset that challenges algorithms to preserve both the precision of periodic trends and the integrity of irregular dynamics, ensuring comprehensive validation for resampling tasks.

### 4.2. Real World Data

In addition to the synthetic dataset, we evaluate our approach on three real-world datasets to validate its practical performance. This ensures a complete assessment across both controlled and realistic scenarios.

#### 4.2.1. PJM Hourly Energy Consumption

**Figure 4:**
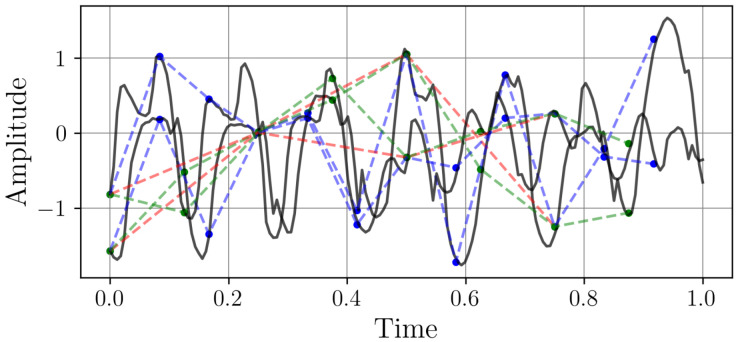
Two samples from the PJM Hourly Energy Consumption dataset with different sampling rates. The time axes represent one week. The continuous black line is the original high-resolution signal ($w_{i}\in R^{168}$). The red, green, and blue markers represent the signal downsampled to resolutions of 4, 8, and 12 points per week, respectively.

This dataset originates from PJM Interconnection, a regional transmission organization responsible for coordinating wholesale electricity transmission across 13 U.S. states. The dataset encompasses hourly electricity consumption values, expressed in MW, spanning the period from 31 December 1998 to 31 December 2001. The dataset is partitioned into *N* non-overlapping weekly segments, each represented as a column vector in the matrix $W\in R^{168\times N}$, such that $W=[w_{1},w_{2},\ldots ,w_{N}]$ where each $w_{i}\in R^{168}$ contains the hourly consumption values for the *i*-th week. Downsampling is applied to each $w_{i}$ at different sampling rates, producing reduced-resolution versions. Let $D_{k}\in R^{m_{k}\times N}$ with *k* = 1, …, 5 denote the downsampled data for the *k*-th sampling frequency, where $m_{k}\in 4,8,12,42$ is the number of samples retained per week. Figure 4 represents two randomly selected samples with different sampling rates.

Both samples exhibit characteristic energy consumption patterns with daily peaks and nightly troughs, reflecting typical grid behavior. This dataset demonstrates how different sampling rates capture varying levels of temporal detail in power systems, from gradual base load changes to rapid demand fluctuations.

#### 4.2.2. Electricity Transformer Temperature

**Figure 5:**
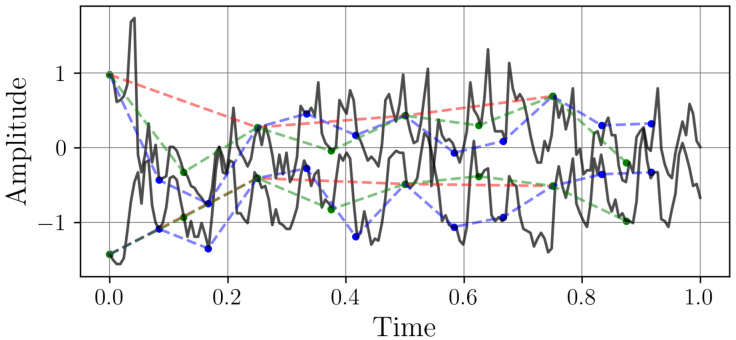
Two samples from the Electricity Transformer Temperature dataset with different sampling rates. The time axes represent one week. The continuous black line is the original high-resolution signal ($w_{i}\in R^{168}$). The red, green, and blue markers represent the signal downsampled to resolutions of 4, 8, and 12 points per week, respectively.

For a broader evaluation, we employed the ETTh1 dataset, a multivariate time series represented as $X\in R^{T\times 7},$ where each row $x_{t}\in R^{7}$ corresponds to an hourly observation of electricity transformer temperature and six power load features at time *t*, and T=17,420 spans the two-year period from July 2016 to July 2018. We focused on the Load Unit Fluctuations per Load (LUFL) variable, denoted as the scalar time series $y=X\left[:,j\right]\in R^{T}$, where *j* is the column index of LUFL in X. Following the same downsampling procedure as applied to the PJM dataset, we partitioned y into weekly segments ${\left\{w_{i}\right\}}_{i=1}^{N}$, each of length 168 (hours/week). For each downsampling frequency $m_{k}\in \left\{4,8,12,42\right\}$ the low resolution version results in $W^{\left(k\right)}=\left[w_{1}^{\left(k\right)},\ldots ,w_{N}^{\left(k\right)}\right]\in R^{m_{k}\times N}$. Figure 5 represents two randomly selected samples with different sampling rates.

Both samples reveal characteristic daily patterns of electricity demand, with the first showing relatively stable daily peaks and the second exhibiting more volatile load fluctuations. The higher sampling rates (particularly 50 Hz) capture crucial rapid transients, sudden demand spikes, and quick ramps, which lower frequencies might miss or distort. This combination of regular patterns and irregular disturbances makes the PJM dataset particularly valuable for developing robust resampling techniques that maintain fidelity across temporal scales.

#### 4.2.3. Thermal Power Prediction

**Figure 6:**
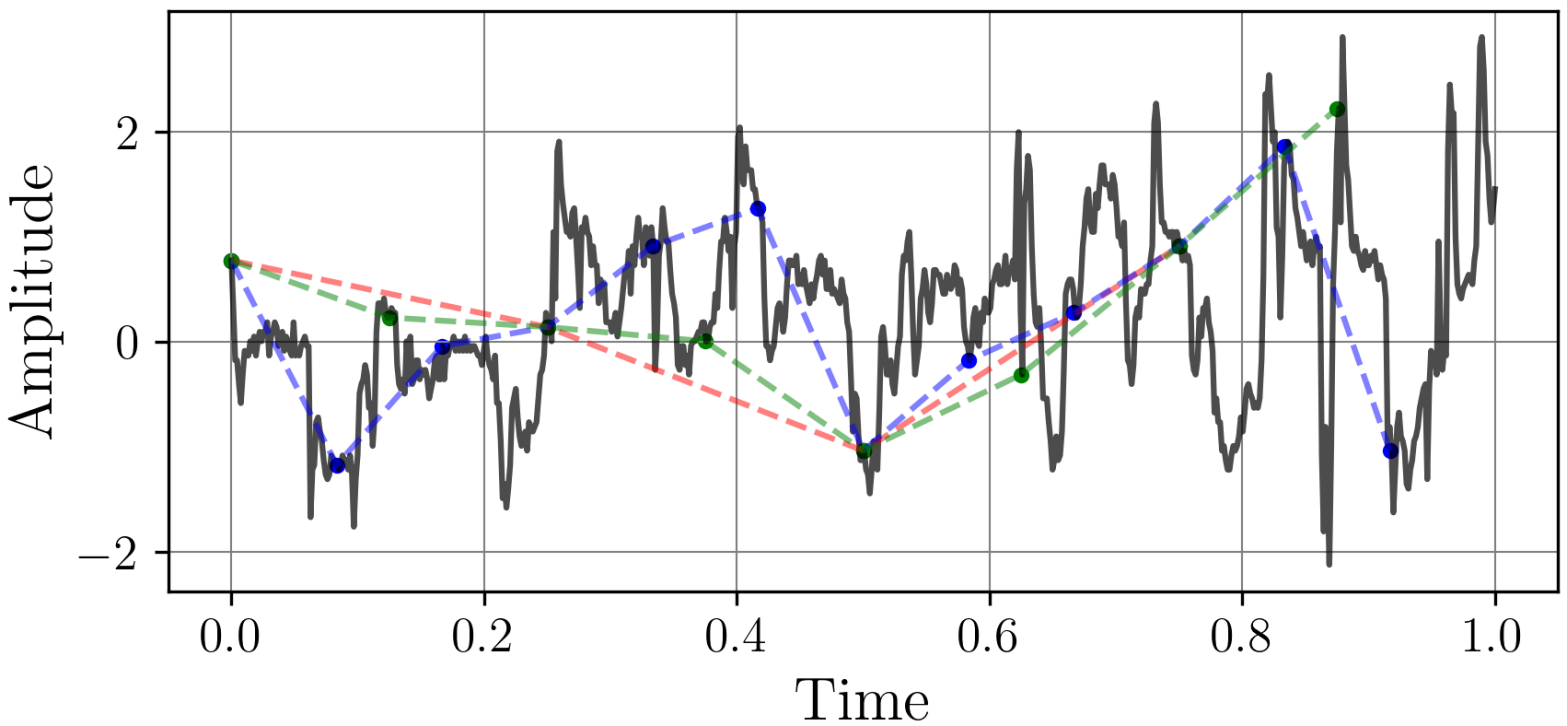
One sample from the Thermal Power Prediction dataset with different sampling rates. The time axes represent one week. The continuous black line is the original high-resolution signal ($w_{i}\in R^{672}$). The red, green, and blue markers represent the signal downsampled to resolutions of 4, 8, and 12 points per week, respectively.

Finally, we incorporated a third real-world dataset to evaluate our methodology on thermal power prediction. The dataset consists of multivariate time series observations recorded at a 15 min sampling frequency from 1 January 2016, to 15 September 2020. Let $Z\in R^{T\times d}$ represent the full dataset, where each row $z_{t}\in R^{d}$ contains measurements at time *t* for *d* variables (including outdoor temperature, flow temperature, return temperature, thermal power output, and water quantity). Here, *T* denotes the total number of 15 min samples over the 4.7-year period. Given the dominant influence of outdoor temperature and thermal power on heat consumption, we extracted the thermal power time series $p=Z\left[:,i\right]\in R^{T}$ where *i* is the column index corresponding to thermal power. We further refined p to focus on winter months, yielding a subset $p_{\mathrm{winter}}\in R^{T^{\prime }}$ with $T^{\prime }<T$. Consistent with prior datasets, we partitioned $p_{\mathrm{winter}}$ into weekly segments ${\left\{w_{i}\right\}}_{i=1}^{N}$, each comprising 672 samples (7 days at 15 min intervals). For each downsampling frequency $m_{k}\in \left\{4,8,12,42\right\}$, the low-resolution version results in $W^{\left(k\right)}=\left[w_{1}^{\left(k\right)},\ldots ,w_{N}^{\left(k\right)}\right]\in R^{m_{k}\times N}$. Figure 6 represents one randomly selected sample with different sampling rates.

This dataset’s value lies in its authentic representation of both gradual thermal processes and sudden operational changes, from slow fuel-based heat accumulation to fast turbine adjustments. The presence of these multi-timescale phenomena makes it particularly suitable for evaluating resampling algorithms’ ability to reconstruct the full spectrum of thermal plant dynamics.

### 4.3. Experimental Setup

We evaluate our proposed MR-BoF model against traditional resampling algorithms, including filter-based methods, and a Feed-Forward Neural Network (FFNN) baseline across one synthetic and three real-world datasets. Our MR-BoF architecture employs a total of nine basis functions. These include three for seasonality (sine, three parameters each), three for trend (linear, two parameters each), and three for events (Gaussian, three parameters each). These functions are distributed among three encoders, each with a $L_{\max}-50-25-10-\dim\left(z_{i}\right)$ structure with ReLU activations between all layers. We test MR-BoF models with one and two stages on the synthetic dataset and a three-stage model on the real-world datasets. As a key baseline, we utilize the MR-BoF’s multi-resolution resampling mechanism but replace the Bag of Functions (BoF) component with an FFNN. This FFNN is designed with a comparable number of trainable parameters for a fair architectural comparison. Specifically, its architecture for the synthetic dataset maps an input of 100 dimensions to an output of 100 dimensions via three hidden layers of 100 neurons each, while for the real-world datasets, it comprises two hidden layers of 168 neurons each between an input and output of 168 dimensions. Both FFNN architectures use ReLU activation functions between all layers. All neural models were trained for $1\times 10^{2}$ epochs using the ADAM optimizer with a batch size of 32, and performance was evaluated after each epoch using the Mean Squared Error (MSE) loss function, L. To ensure a robust comparison and account for variability in training dynamics, this training procedure was repeated 10 times for each model on each dataset.

### 4.4. Results

**Figure 7:**
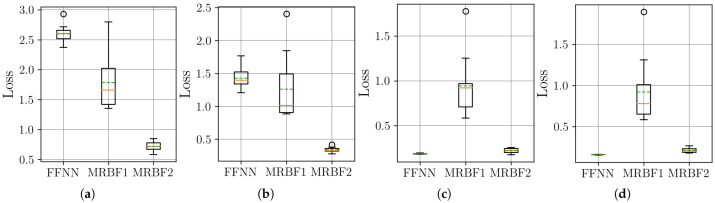
Distribution of the final MSE loss on the test set over 10 independent runs for the neural network models. Within each box, the solid orange line and dashed green line denote the median and the mean, respectively. The boxplots compare the performance of the FFNN, single-stage MR-BoF (MRBF1), and two-stage MR-BoF (MRBF2) across four scenarios: (**a**) 10 Hz to 100 Hz. (**b**) 20 Hz to 100 Hz. (**c**) 50 Hz to 100 Hz. (**d**) 500 Hz to 100 Hz.

In this section, we examine the effectiveness of time series decomposition and reconstruction using the MR-BoF framework with multi-resolution input sequences and compare it to the performance obtained using traditional resampling algorithms and an FFNN baseline. Therefore, to systematically evaluate our approach under varying sampling conditions, we design four distinct resampling scenarios: three upsampling cases (from 10 Hz, 20 Hz, and 50 Hz to a target rate of 100 Hz) and one downsampling case (from 500 Hz to 100 Hz). This framework allows us to assess performance across a wide spectrum of temporal resolutions, ranging from highly sparse to oversampled data, ensuring robustness in both interpolation and decimation tasks. We begin by analyzing the stability of the neural network models on the synthetic dataset. The distribution of the final MSE loss on the test set, obtained over 10 independent training runs for each model, is visualized using boxplots in Figure 7.

**Figure 8:**
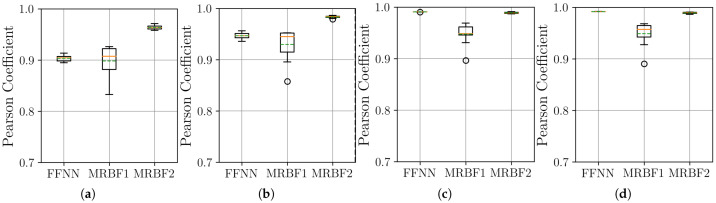
Distribution of the Pearson coefficient on the test set over 10 independent runs. Within each box, the solid orange and dashed green lines denote the median and the mean, respectively. The results are shown across four scenarios: (**a**) 10 Hz to 100 Hz. (**b**) 20 Hz to 100 Hz. (**c**) 50 Hz to 100 Hz. (**d**) 500 Hz to 100 Hz.

The boxplots in Figure 7 show that the MR-BoF models significantly outperform the FFNN baseline in the high-ratio upsampling scenarios (a, b). Notably, the single-stage MR-BoF 1 achieves this with approximately half the parameters of the FFNN, while the two-stage MR-BoF 2, with a comparable parameter count, demonstrates the strongest performance. In the less demanding scenarios with higher sampling frequencies (c, d), the performance of the models converges, with both the FFNN and the two-stage MR-BoF achieving low and comparable reconstruction errors. This indicates that the MR-BoF architecture provides a more robust solution overall, especially for challenging reconstruction tasks. To complement this error-based analysis, we next evaluate the models using the Pearson correlation coefficient, shown in Figure 8.

The Pearson correlation results in Figure 8 corroborate the findings from the MSE analysis. In the challenging upsampling cases (a, b), the MR-BoF models achieve median coefficients closer to 1 and with significantly less variance than the FFNN. For scenarios (c) and (d), all models perform quite well, with coefficients near unity. Taken together, the MSE and Pearson results indicate that the MR-BoF framework not only minimizes reconstruction error but also preserves the structural correlation of the time series more effectively, especially in demanding, low-resolution scenarios.

To facilitate a direct numerical comparison against the deterministic traditional algorithms, we selected the best-performing trial from the 10 independent runs for each neural network. The results of this head-to-head comparison are detailed in Table 1, which presents both Mean Squared Error and Pearson Correlation for all methods across the resampling scenarios.

**Figure 9:**
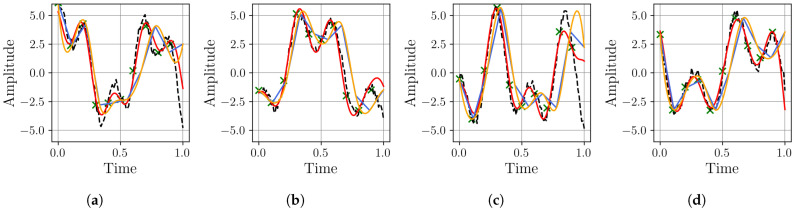
Resampling performance comparison for the synthetic dataset: Our proposed BoF approach (red) versus linear (blue) and cubic (yellow) interpolation methods for upsampling an input signal with a sampling rate of 10 Hz (green points) to a target sampling rate of 100 Hz (dashed black line). Subfigures (**a**–**d**) show four different samples of this process.

The results in Table 1 confirm the superiority of the two-stage MR-BoF model in upsampling scenarios. For the most challenging 10 Hz case, it achieves an MSE of 0.682, surpassing the best traditional method (Polyphase FIR, 0.882). This trend continues for the 20 Hz and 50 Hz cases. In the downsampling scenario (500 Hz), however, the classic filter-based methods exhibit a marginally lower MSE. This suggests that while MR-BoF provides a dominant solution for resampling from sparse data, conventional filters remain highly effective for decimation tasks where sufficient data is available. To validate this further, we present four resampled signals in Figure 9.

**Figure 10:**
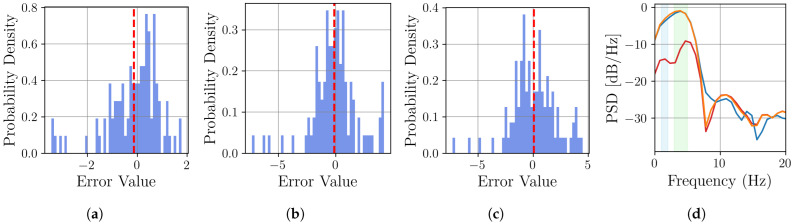
Comparative analysis of the reconstruction error. (**a**–**c**) Time-domain error distributions for the MR-BoF model (μ=−0.12,σ=1.03), linear interpolation (μ=−0.13,σ=1.98), and cubic interpolation (μ=0.07,σ=2.02). The red dashed line indicates the mean error. (**d**) Power Spectral Density (PSD) comparison of the error in the resampling methods MR-BoF (red), linear (blue), and cubic (yellow). Shaded regions highlight the primary seasonal frequency bands (0–2 Hz and 5–8 Hz).

The visual results in Figure 9 highlight the distinct behaviors of each resampling method. Linear interpolation inherently produces sharp, angular reconstructions, while cubic interpolation, although smoother, introduces oscillatory artifacts leading to significant overshoots and undershoots. The proposed MR-BoF model successfully avoids both of these issues, tracking the ground truth signal with more fidelity. This synthetic dataset is particularly insightful for such a comparison, as its well-defined composition allows for a targeted evaluation of how each model handles specific, known frequency components, such as seasonality. To quantify these observations, we perform a detailed error analysis on the first representative sample, shown in panel (a) of Figure 9. The results, presented in Figure 10, confirm the superiority of the proposed model by revealing its significantly lower error variance and spectral power in critical frequency bands.

The quantitative error analysis presented in Figure 10 confirms the superiority of the MR-BoF model. While a time-domain analysis shows that all methods are effectively unbiased with a mean error closed to zero, MR-BoF exhibits a significantly lower standard deviation than its counterparts, indicating a more consistently precise reconstruction.

However, the primary advantage of the MR-BoF approach is most evident in the frequency-domain analysis (Figure 10d). Within the critical seasonal frequency bands, MR-BoF achieves a drastic reduction in error power. In the 1–2 Hz band, its average spectral error is −14.0 dB, outperforming linear and cubic interpolation by more than 10 dB. This substantial advantage is mirrored in the 3–5 Hz band, where our model is again over 10.5 dB better than the baseline methods. It is crucial to note that a 10 dB improvement corresponds to a tenfold reduction in error power, underscoring the model’s advanced capability to faithfully reconstruct fundamental signal components where traditional methods fail.

Similarly, we evaluate the performance of our approach on the real-world PJM Hourly Energy Consumption dataset. Unlike synthetic or controlled datasets, real-world data introduces challenges such as irregular sampling rates, diverse sensor noises, and environmental disturbances, making it a crucial benchmark for assessing the robustness of our method.

**Figure 11:**
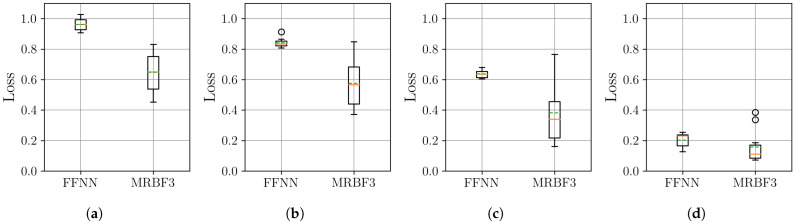
Distribution of the final MSE loss on the PJM Hourly Energy Consumption dataset, with over 10 independent runs for the neural network models. Within each box, the solid orange line and dashed green line denote the median and the mean, respectively. The boxplots compare the performance of the FFNN, and three-stage MR-BoF (MRBF3) across four scenarios: (**a**) $R^{4}\to R^{168}$. (**b**) $R^{8}\to R^{168}$. (**c**) $R^{12}\to R^{168}$. (**d**) $R^{42}\to R^{168}$.

Since downsampling poses no significant challenge compared to upsampling, we focus our evaluation on the more demanding upsampling tasks using real-world datasets. Specifically, we examine four progressively difficult scenarios: from extremely sparse signals ($R^{4}$) through intermediate sampling rates ($R^{8}$, $R^{12}$ ) to relatively dense signals ($R^{42}$), all targeting a final sampling rate of $R^{168}$. This range allows us to test our method’s performance across varying degrees of signal sparsity, from near-critical sampling conditions to more favorable cases. Following the methodology of 10 independent training runs per model, the resulting distributions for MSE are presented in Figure 11.

**Figure 12:**
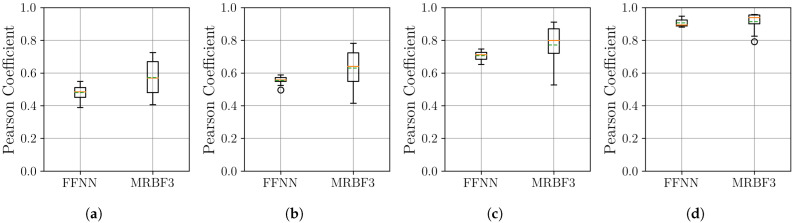
Distribution of the Pearson coefficient on the PJM Hourly Energy Consumption dataset, with over 10 independent runs for the neural network models. Within each box, the solid orange line and dashed green line denote the median and the mean, respectively. The boxplots compare the performance of the FFNN, and three-stage MR-BoF (MRBF3) across four scenarios: (**a**) $R^{4}\to R^{168}$. (**b**) $R^{8}\to R^{168}$. (**c**) $R^{12}\to R^{168}$. (**d**) $R^{42}\to R^{168}$.

The results from the PJM dataset confirm the effectiveness of the MR-BoF architecture for real-world applications. The MSE boxplots in Figure 11 show that the three-stage MR-BoF model (MRBF3) achieves a consistently lower median loss than the FFNN baseline across all four sparsity levels. The performance advantage is most pronounced in the extremely low-sampled scenario (**a**), underscoring the model’s strength in low-sampling-rate regimes. To complement this error-based analysis, we next examine the Pearson correlation coefficient results, shown in Figure 12.

The previous conclusion is reinforced by the Pearson correlation results in Figure 12, where the MR-BoF model again demonstrates superior performance. Across all four scenarios, the MR-BoF achieves a higher median coefficient than the FFNN, indicating a better reconstruction of the signal’s structural properties. A key observation relates to stability: while the MR-BoF exhibits high variance in the most challenging sparse scenarios (a, b), its performance becomes progressively more stable as the input signal density increases, achieving low variance in the densest case (d). Taken together, these results demonstrate the robustness of the MR-BoF framework for demanding, real-world upsampling tasks, confirming its ability to both minimize error and preserve signal correlation more effectively than a standard neural network baseline. To provide a direct numerical comparison for the PJM dataset, we selected the best-performing neural models from the 10 independent runs. The results of this comparison against the deterministic traditional algorithms are detailed in Table 2.

The results presented in Table 2 demonstrate how each approach adapts to real-world conditions. Consistent with our findings on the synthetic data, the MR-BoF model shows superior performance in the most critical scenarios involving very sparse inputs ($R^{4}$ to $R^{12}$), achieving both a significantly lower MSE and a higher Pearson coefficient than all baselines. However, in the high-density scenario ($R^{42}$), where signal information is abundant, traditional frequency-domain methods like FFT-based resampling achieve the best reconstruction accuracy. This is likely due to their ability to effectively exploit the signal’s spectral properties when a sufficient number of samples is available. Nevertheless, our approach remains competitive, yielding substantially better MSE and Pearson scores than cubic and linear interpolation.

**Figure 13:**
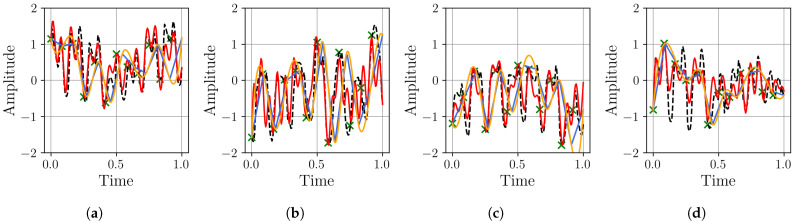
Resampling performance comparison for the PJM Hourly Energy Consumption dataset: Our proposed BoF approach (red) versus linear (blue) and cubic (yellow) interpolation methods for upsampling a $R^{12}$ input signal (green points) to a target $R^{168}$ sampling rate (dashed black line). Subfigures (**a**–**d**) show four different samples of this process.

To provide a qualitative illustration of these performance differences, Figure 13 visually compares the resampling results of three methods for the challenging $R^{12}\to R^{168}$ scenario.

The BoF approach demonstrates clear advantages in handling this complex, real-world dataset. While linear interpolation produces abrupt, piecewise transitions that distort consumption patterns, and cubic interpolation creates unrealistic fluctuations in energy trends, the BoF method maintains smoother, more physically plausible trajectories. It better captures the underlying consumption behavior without introducing artificial peaks or troughs.

**Figure 14:**
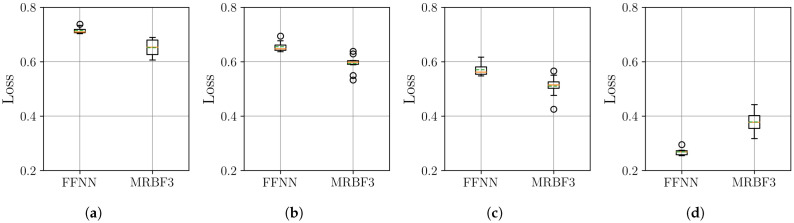
Distribution of the final MSE loss on the Electricity Transformer Temperature dataset, with over 10 independent runs for the neural network models. Within each box, the solid orange line and dashed green line denote the median and the mean, respectively. The boxplots compare the performance of the FFNN, and three-stage MR-BoF (MRBF3) across four scenarios: (**a**) $R^{4}\to R^{168}$. (**b**) $R^{8}\to R^{168}$. (**c**) $R^{12}\to R^{168}$. (**d**) $R^{42}\to R^{168}$.

**Figure 15:**
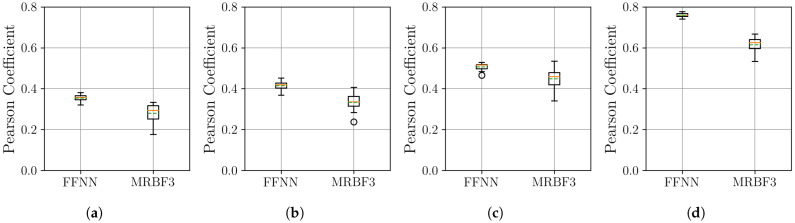
Distribution of the Pearson coefficient on the Electricity Transformer Temperature dataset, with over 10 independent runs for the neural network models. Within each box, the solid orange line and dashed green line denote the median and the mean, respectively. The boxplots compare the performance of the FFNN, and three-stage MR-BoF (MRBF3) across four scenarios: (**a**) $R^{4}\to R^{168}$. (**b**) $R^{8}\to R^{168}$. (**c**) $R^{12}\to R^{168}$. (**d**) $R^{42}\to R^{168}$.

To test the generalization of our method on time series with different characteristics, we next evaluate it on the Electricity Transformer Temperature dataset. Following the same experimental protocol of 10 independent runs, the performance distributions for MSE and Pearson correlation are presented in Figure 14 and Figure 15, respectively.

The results on the Electricity Transformer Temperature dataset highlight the adaptability of the MR-BoF framework. The MSE analysis in Figure 14 confirms that our model is the superior choice for minimizing reconstruction error in the critical scenarios (a, b, c). While the simpler FFNN baseline is highly competitive in the highest-resolution case (d) and across the Pearson correlation results (Figure 15), this dynamic provides a key insight. It demonstrates that the MR-BoF’s performance remains robust and comparable to standard deep learning baselines even on datasets where its specialized inductive biases may be less critical.

Building on the insights from the boxplots, Table 3 provides a direct numerical comparison, benchmarking the best-performing trial from each neural network against the deterministic traditional algorithms.

**Figure 16:**
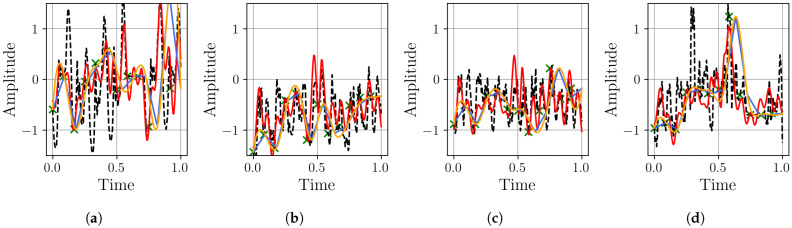
Resampling performance comparison for the Electricity Transformer Temperature dataset: Our proposed BoF approach (red) versus linear (blue) and cubic (yellow) interpolation methods for upsampling a $R^{12}$ input signal (green points) to a target $R^{168}$ sampling rate (dashed black line). Subfigures (**a**–**d**) show four different samples of this process.

Mirroring our previous results, the MR-BoF approach again shows dominant performance on the Electricity Transformer Temperature dataset for sparse sampling regimes ($R^{4}$–$R^{12}$), overcoming the signal reconstruction challenges that limit conventional methods in low-data conditions. Conversely, in energy systems operating at high sampling densities ($R^{42}$), spectral methods maintain their advantage. This consistent image of our core findings across domains confirms the BoF method’s generalizability for sparse time series reconstruction. Figure 16 visually compares the reconstruction quality of our MR-BoF method against two baseline approaches (linear interpolation and cubic spline) for upsampling $R^{12}$ to $R^{168}$.

The visual results in Figure 16 confirm the superiority of the BoF approach in handling this complex, real-world dataset. While linear interpolation produces abrupt, piecewise transitions that distort consumption patterns, and cubic interpolation creates unrealistic fluctuations, the BoF method maintains smoother, more physically plausible trajectories. It better captures the underlying consumption behavior without introducing artificial peaks or troughs, which is crucial for downstream tasks such as thermal monitoring and predictive maintenance.

**Figure 17:**
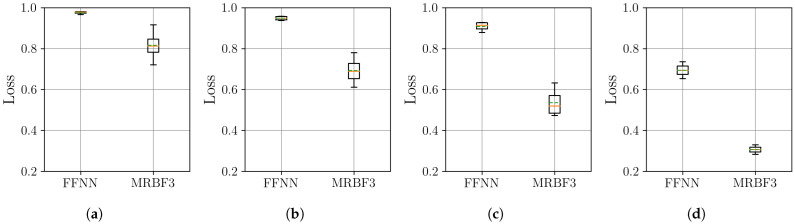
Distribution of the final MSE loss on the Thermal Power Prediction dataset, with over 10 independent runs for the neural network models. Within each box, the solid orange line and dashed green line denote the median and the mean, respectively. The boxplots compare the performance of the FFNN, and three-stage MR-BoF (MRBF3) across four scenarios: (**a**) $R^{4}\to R^{168}$. (**b**) $R^{8}\to R^{168}$. (**c**) $R^{12}\to R^{168}$. (**d**) $R^{42}\to R^{168}$.

As a final validation, we assess the framework’s performance on the Thermal Power Prediction dataset, which contains heat demand data from district heating systems in Germany. This evaluation introduces distinct real-world complexities, including strong daily variability and abrupt demand shifts caused by weather fronts, providing a challenging test for the model’s adaptability. First, we examine the stability and performance of the neural networks using the MSE metric, with the results from 10 independent runs shown in Figure 17.

**Figure 18:**
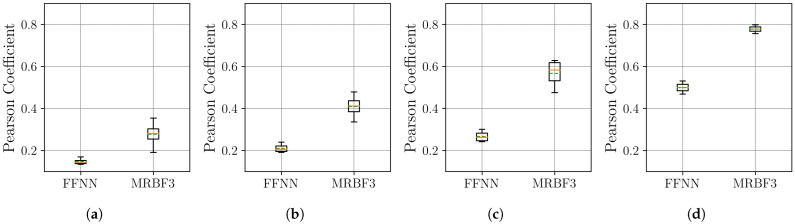
Distribution of the Pearson coefficient on the Thermal Power Prediction dataset, with over 10 independent runs for the neural network models. Within each box, the solid orange line and dashed green line denote the median and the mean, respectively. The boxplots compare the performance of the FFNN, and three-stage MR-BoF (MRBF3) across four scenarios: (**a**) $R^{4}\to R^{168}$. (**b**) $R^{8}\to R^{168}$. (**c**) $R^{12}\to R^{168}$. (**d**) $R^{42}\to R^{168}$.

The MSE results in Figure 17 show a clear performance advantage for the MR-BoF architecture on this dataset. In all four scenarios, the MR-BoF achieves a significantly lower median loss than the FFNN. Notably, the entire distribution of the MR-BoF’s performance is superior; its 75th percentile for loss is consistently lower than the FFNN’s 25th percentile. This indicates that even the worst-performing MR-BoF trials yielded more accurate results than the best-performing FFNN trials, confirming the framework’s robust advantage for this task. These findings are corroborated by the Pearson correlation analysis shown in Figure 18.

The Pearson correlation analysis in Figure 18 reinforces the superiority of the MR-BoF model. Across all four scenarios, the MR-BoF consistently achieves a substantially higher median Pearson coefficient, indicating a far better reconstruction of the signal’s structural properties. Based on the preceding analysis, we selected the best trial for the MR-BoF model to benchmark it against the traditional algorithms. These results are detailed in Table 4.

**Figure 19:**
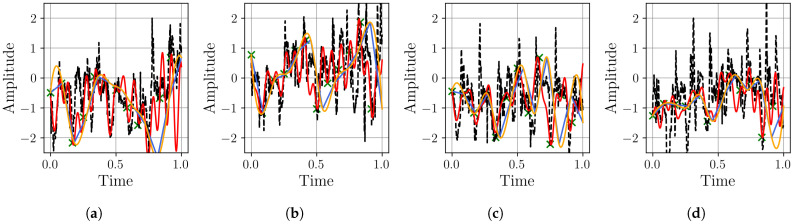
Resampling performance comparison for the Thermal Power Prediction dataset: Our proposed BoF approach (red) versus linear (blue) and cubic (yellow) interpolation methods for upsampling a $R^{12}$ input signal (green points) to a target $R^{168}$ sampling rate (dashed black line). Subfigures (**a**–**d**) show four different samples of this process.

The results demonstrate MR-BoF’s superior performance in resampling thermal power data, particularly for low resolution inputs ($R^{4}$–$R^{12}$) where it achieves significantly lower MSE (0.802–0.488) and higher correlation (up to 0.614) than conventional methods. While filter-based approaches perform well at high resolution ($R^{42}$), MR-BoF maintains competitive accuracy while excelling where it matters most, the challenging low-resolution cases. Traditional linear and cubic interpolation show poor correlation and higher errors, especially their extended variants. Fourier methods, though reasonable at high resolution, fail to match MR-BoF’s performance with sparse data. This advantage stems from MR-BoF’s multi-scale feature learning capability, which better captures the complex temporal dynamics compared to fixed mathematical interpolations or frequency-domain approaches. Figure 19 visually compares the upsampling ($R^{12}$→$R^{168}$) results of our method against two baseline approaches.

These visualizations confirm the quantitative results, showing how the MR-BoF method maintains smoother, more plausible trajectories that better capture the underlying demand behavior without the jagged segments or unrealistic fluctuations produced by traditional methods. This analysis highlights how the optimal resampling strategy depends on the signal’s sampling density, with our MR-BoF method proving particularly valuable for challenging, low-resolution reconstruction tasks.

### 4.5. Discussion and Limitations

The proposed Multi-Resolution Bag of Functions framework provides a novel and effective approach to modeling time series with heterogeneous sampling rates. To comprehensively evaluate the approach, we discuss challenges and limitations, which in turn delineate directions for future research.

A foundational challenge lies in the selection of an appropriate basis function set. In scenarios where domain-specific knowledge is scarce, the choice of functions may become arbitrary, potentially leading to a sub-optimal representation that compromises model efficiency. However, we also note that the choice of seasonality, trend, and event functions is quite straightforward and typically requires low domain-specific knowledge.

Further, we discuss the framework’s computational cost. Using the synthetic dataset detailed in Section 4.1, we benchmarked the execution time of the same models whose performance was previously evaluated in Table 1. The results of this computational analysis are summarized in Table 5.

All performance benchmarks were conducted on a workstation equipped with an Intel Core i7-11700 CPU, 64 GB of RAM, and an NVIDIA RTX 5000 GPU. The software stack included PyTorch 1.12.1+cu113 for neural models and SciPy 1.13.1 for traditional methods. As noted in Table 5, neural models utilized GPU acceleration, while DSP algorithms were executed on the CPU. Each reported value is the average of 10 independent runs on fixed-length signals with a batch size of one.

As expected, the benchmark results highlight the significant computational cost of the MR-BoF architecture compared to conventional methods. Specifically, the single-stage MR-BoF 1 is much slower than highly efficient FFT-based resampling (approx. 940 ms vs. 20 ms). This contrasts sharply with traditional DSP algorithms, whose performance is either consistently fast (cubic interpolation) or predictably dependent on signal properties like the resampling ratio (FIR filter). However, it should be noted that the higher computational cost does not pose any restriction for the practical usability.

A particularly instructive comparison can be made with the FFNN, which has a comparable number of trainable parameters. The FFNN is faster than our models due to the highly optimized matrix multiplications in modern deep learning hardware. In contrast, the MR-BoF’s processing time is not optimized to date and is driven by the evaluation of basis functions. Hence, while the challenge of basis function selection and the associated computational costs are important considerations, they also define clear pathways for optimization. Potential strategies include developing methods for sparse basis function evaluation, model quantization, or knowledge distillation.

## 5. Conclusions

In this work, we presented a novel Bag of Functions framework for the adaptive decomposition and reconstruction of time series data with heterogeneous sampling rates. By transforming signals into a continuous functional representation, our approach overcomes key limitations of traditional interpolation and filtering methods, eliminating spectral distortions while enabling flexible handling of sparse and dense sampling regimes. Our evaluation demonstrates three key advances: (1) the BoF model achieves lower MSE and higher Pearson correlation than conventional methods in critical sparse-sampling scenarios, (2) validation across diverse datasets confirms consistent performance under diverse real-world conditions, and (3) the staged learning architecture yields lower output variance, proving especially valuable for industrial applications requiring reliable sparse-data recovery. Overall, this work introduces a sampling-rate-agnostic framework for time series modeling, offering a new paradigm with broad applicability in smart infrastructure, IoT systems, and beyond.

## Figures and Tables

**Table 1:** Comparison of the performance of resampling methods from different sampling frequencies up to the target sampling frequency of 100 Hz using MSE ↓ and Pearson Correlation ↑ on the synthetic dataset. The best results for each metric are shown in bold.

Method	MSE ↓				Pearson ↑			
	10 Hz	20 Hz	50 Hz	500 Hz	10 Hz	20 Hz	50 Hz	500 Hz
Linear Interpol.	4.450	1.336	0.224	0.189	0.731	0.925	0.987	0.989
Cubic Interpol.	5.553	1.440	0.242	0.206	0.692	0.922	0.986	0.988
FFT-based	1.531	0.526	0.244	0.142	0.918	0.972	0.986	0.992
Polyphase FIR	0.882	0.357	0.205	0.118	0.952	0.980	0.988	**0.993**
FIR Filter	0.933	0.349	0.200	**0.117**	0.949	0.981	0.989	**0.993**
Sinc Filter	1.086	0.348	0.201	**0.117**	0.940	0.981	0.989	**0.993**
FFNN	2.371	1.207	0.188	0.157	0.913	0.956	0.990	0.991
MR-BoF 1 Stage	1.180	0.638	0.442	0.423	0.939	0.967	0.977	0.978
MR-BoF 2 Stage	**0.682**	**0.327**	**0.175**	0.183	**0.965**	**0.984**	**0.991**	0.990

**Table 2:** Comparison of the performance of resampling methods from different sampling frequencies up to the target $R^{m_{k}}\to R^{168}$ with $m_{k}\in \left\{4,8,12,42\right\}$ using MSE ↓ and Pearson Correlation ↑ on the PJM Hourly Energy Consumption dataset. The best results for each metric are shown in bold.

Method	MSE ↓				Pearson ↑			
	$R^{4}$	$R^{8}$	$R^{12}$	$R^{42}$	$R^{4}$	$R^{8}$	$R^{12}$	$R^{42}$
Linear Interpol.	1.352	1.345	0.939	0.186	0.070	0.116	0.308	0.879
Linear Interpol. ext.	1.569	1.299	0.895	0.056	0.153	0.216	0.432	0.963
Cubic Interpol.	1.598	1.434	1.114	0.201	0.077	0.122	0.292	0.874
Cubic Interpol. ext.	3.753	1.809	5.537	0.131	0.191	0.197	0.121	0.919
FFT-based	1.365	1.345	1.233	**0.026**	0.160	0.186	0.297	**0.983**
Polyphase FIR	1.263	1.295	1.129	0.033	0.195	0.199	0.325	0.978
FIR Filter	1.255	1.289	0.984	0.032	0.193	0.199	0.376	0.978
Sinc Filter	1.247	1.290	1.112	0.033	0.192	0.196	0.312	0.978
FFNN	0.979	0.816	0.611	0.131	0.471	0.587	0.736	0.941
MR-BoF 3 Stages	**0.451**	**0.370**	**0.160**	0.072	**0.724**	**0.781**	**0.911**	0.957

**Table 3:** Comparison of the performance of resampling methods from different sampling frequencies up to the target $R^{m_{k}}\to R^{168}$ with $m_{k}\in \left\{4,8,12,42\right\}$ using MSE ↓ and Pearson Correlation ↑ on the Electricity Transformer Temperature dataset. The best results for each metric are shown in bold.

Method	MSE ↓				Pearson ↑			
	$R^{4}$	$R^{8}$	$R^{12}$	$R^{42}$	$R^{4}$	$R^{8}$	$R^{12}$	$R^{42}$
Linear Interpol.	0.736	0.760	0.680	0.237	0.076	0.069	0.181	0.736
Linear Interpol. ext.	1.372	0.936	0.540	0.235	0.039	0.159	0.349	0.754
Cubic Interpol.	0.729	0.872	0.803	0.264	0.108	0.058	0.166	0.727
Cubic Interpol. ext.	4.046	3.304	2.710	0.305	0.032	0.110	0.169	0.723
FFT-based	0.924	0.801	0.695	0.223	0.061	0.179	0.274	0.786
Polyphase FIR	0.885	0.745	0.643	0.217	0.084	0.211	0.305	0.789
FIR Filter	0.867	0.738	0.617	**0.214**	0.085	0.208	0.318	**0.790**
Sinc Filter	0.847	0.737	0.639	0.217	0.079	0.193	0.289	0.786
FFNN	0.702	0.639	0.547	0.270	**0.345**	**0.415**	0.515	0.760
MR-BoF 3 Stages	**0.606**	**0.532**	**0.424**	0.317	0.320	0.378	**0.534**	0.667

**Table 4:** Comparison of the performance of resampling methods from different sampling rates up to the target $R^{m_{k}}\to R^{168}$ with $m_{k}\in \left\{4,8,12,42\right\}$ using MSE ↓ and Pearson Correlation ↑ on the Thermal Power Prediction dataset. The best results for each metric are shown in bold.

Method	MSE ↓				Pearson ↑			
	$R^{4}$	$R^{8}$	$R^{12}$	$R^{42}$	$R^{4}$	$R^{8}$	$R^{12}$	$R^{42}$
Linear Interpol.	1.021	1.021	0.939	0.497	0.145	0.205	0.255	0.623
Linear Interpol. ext.	1.352	1.068	0.909	0.285	0.182	0.247	0.340	0.796
Cubic Interpol.	1.221	1.170	1.075	0.567	0.127	0.192	0.243	0.607
Cubic Interpol. ext.	5.484	2.107	2.578	0.570	0.115	0.201	0.165	0.721
FFT-based	1.105	1.128	1.094	0.295	0.186	0.222	0.258	0.804
Polyphase FIR	1.055	1.087	1.038	0.287	0.212	0.235	0.278	0.807
FIR Filter	1.036	1.076	0.991	**0.280**	0.214	0.236	0.297	**0.811**
Sinc Filter	1.001	1.072	1.020	0.290	0.217	0.235	0.273	0.803
FFNN	0.965	0.937	0.878	0.600	0.168	0.239	0.300	0.572
MR-BoF 3 Stages	**0.802**	**0.667**	**0.488**	0.282	**0.285**	**0.422**	**0.614**	0.797

**Table 5:** Mean execution time (ms) and standard deviation for resampling signals to 100 Hz. The proposed MR-BoF models are compared against traditional DSP algorithms and a feed-forward neural network-based baseline. GPU-accelerated methods are marked with an asterisk (*).

Input *f* (Hz)	Cubic	FFT	FIR	FFNN *	MR-BoF 1 *	MR-BoF 2 *
10 Hz	60 ± 4	20 ± 1	290 ± 20	170 ± 220	940 ± 227	1780 ± 216
20 Hz	50 ± 2	20 ± 1	170 ± 13	160 ± 225	950 ± 216	1790 ± 224
50 Hz	50 ± 1	20 ± 1	100 ± 11	160 ± 214	940 ± 227	1770 ± 227
500 Hz	50 ± 4	20 ± 1	70 ± 9	160 ± 224	950 ± 219	1770 ± 225

## Data Availability

The original data presented in the study are openly available in PJM Data Miner 2 at https://dataminer2.pjm.com/feed/hrl_load_metered/definition (accessed on 10 January 2025) (PJM Hourly Energy Consumption) and in the ETDataset GitHub Repository at https://github.com/zhouhaoyi/ETDataset (accessed on 10 January 2025) (Electricity Transformer Temperature).
